# Integrating Pharmacology and Microbial Network Analysis with Experimental Validation to Reveal the Mechanism of Composite Sophora Colon-Soluble Capsule against Ulcerative Colitis

**DOI:** 10.1155/2020/9521073

**Published:** 2020-04-20

**Authors:** Yuxuan Ding, Mingjun Chen, Qiying Wang, Lu Gao, Yang Feng, Shida Wang, Zitian Song, Zhanqi Tong

**Affiliations:** ^1^Department of Traditional Chinese Medicine and Acupuncture, The Second Medical Centre, Chinese People's Liberation Army General Hospital, National Clinical Research Center for Geriatric Diseases, Beijing 100853, China; ^2^The Graduate School of Chinese PLA General Hospital, Beijing 100853, China; ^3^Department of Medical Branch, Chinese People's Liberation Army General Hospital, Beijing 100853, China; ^4^Institute of Military Cognition and Brain Sciences, Academy of Military Medical Sciences, Academy of Military Sciences, Beijing 100850, China

## Abstract

Ulcerative colitis (UC) has multifactorial pathogenesis that acts synergistically, such as immune system dysregulation and expansion of infectious gut microbiota. Therefore, a multicomponent treatment derived from Chinese herbal medicine that interacts with multiple targets synergistically is needed. Composite sophora colon-soluble capsule (CSCC) is a Chinese herbal formula that has shown therapeutic efficacy against UC in randomized clinical trials. However, its bioactive components and potential target genes against UC remain unclear. Here, we used a network pharmacology approach to detect component-target-pathway interactions of CSCC against UC. A total of 29 gene targets, 91 bioactive components, and 20 enriched pathways of CSCC were identified. The IL-17 signaling pathway activated by infectious gastrointestinal microbes and predicted by the network analysis to be a major pathway modulated by CSCC against UC was studied in a dextran sulfate sodium-induced colitis model. CSCC showed remarkable efficacy against UC with respect to the attenuation of colon length, body weight loss, and disease activity index through gut microbiota recovery and intestinal immune homeostasis. The rectal administration of CSCC reduced the numbers of Th17 cells isolated from both mesenteric lymph nodes and lamina propria mononuclear cells and the levels of IL-17A, IL-6, IL-1*β*, and TNF-*α*. Additionally, the percentage of Treg cells and the levels of their hallmark cytokines were upregulated. Rectal administration of CSCC led to microbiota regulation with a significant correlation between suppression of Verrucomicrobiaceae and Ruminococcaceae, as well as the elevation of Lactobacillaceae, and CSCC administration via microbiome correlation heatmaps and cooccurrence network analysis at multiple time points. Thus, our study presents an effective herbal formula, CSCC, for UC treatment and explores its components and mechanisms of efficacy through the examination of gut microbiota and hallmark cytokines in the IL-17 pathway.

## 1. Introduction

Ulcerative colitis (UC) is a major inflammatory disorder of the gastrointestinal tract that is characterized by chronic intestinal inflammation that manifests as abdominal pain, rectal bleeding, and diarrhea [1, 2]. The pathogenesis of UC is linked to both complex genes and multiple pathways such as the expansion of gastrointestinal infectious microbiota, damage to the epithelial barrier, and aggravation of associated proinflammatory genetic factors [3, 4]. Drugs used for decades for UC treatment such as 5-aminosalicylic acid (5-ASA), corticosteroids, and antitumor necrosis factor-alpha (TNF-*α*) monoclonal antibody, as well as fecal microbiota transplantation, have only partly improved the clinical outcomes for UC patients. However, such therapies are not particularly effective for the treatment of intractable UC with episodic acute flare-ups, in which multiple proinflammatory cytokines, gut flora, and the regulation of mucosal integrity are disturbed [5–7]. Therefore, a treatment that interacts with multiple targets synergistically is needed for UC.

Chinese herbal medicine contains multiple components and has several target genes, which has aroused much interest in its clinical efficacy for UC treatment [8, 9]. Composite sophora colon-soluble capsule (CSCC) is a Chinese herbal formula drug developed using the OS/Local Colon Medication (OLCM) technique for UC treatment in China. There are five herbs in CSCC: Radix Sophorae Flavescentis (SFR, Ku-Shen in Chinese), Indigo Naturalis (IN, Qing-Dai in Chinese), *Bletilla striata* (BS, Bai-ji in Chinese), Radix Sanguisorbae (RS, Di-yu in Chinese), and Licorice Root (LR, Gan-cao in Chinese). In our previous clinical study comparing CSCC with mesalazine enteric-coated tablets, CSCC exerted a similar comprehensive effect in the treatment of UC, leading to improvement in patients with inflammation in the left hemicolon [10, 11]. The bioactive components and potential target genes that are involved in the effects of CSCC against UC remain unclear.

The complex components of herbal formula interactions for the discovery of treatment mechanisms can be best characterized by the construction of component-target (C-T) networks. Topology analysis can help to understand the key biological functions of target genes and pathways related to a particular herbal formula from the interconnected, complex, biological networks for the relevant disease [12, 13]. Additionally, the interactions between the microbiome and diseases due to the complex mutual association within the microbial community could be identified using network approaches [14, 15]. In this study, we aimed to evaluate the therapeutic efficacy of CSCC in UC and perform network pharmacology and microbiome analyses for the investigation of the interassociation target genes and pathways. We also sought experimental validation of the importance of the interleukin-17 (IL-17) signaling pathway in UC, as predicted by our network results.

## 2. Methods

### 2.1. Network Pharmacology-Based Analysis

#### 2.1.1. CSCC Component Identification

All constituents in the 5 herbal components of CSCC, namely, SFR, IN, BS, RS, and LR, were retrieved from the traditional Chinese medicine (TCM) systems pharmacology (TCMSP) database (http://tcmspw.com/) [16]. Bioactive candidates that could cross the intestinal epithelium barrier in absorption, distribution, metabolism, and excretion (ADME) processes were selected on the basis of three important properties including drug-likeness (DL) index ≥ 0.18, oral bioavailability (OB) ≥ 30%, and intestinal epithelial permeability (in Caco-2 cells) ≥ 0.4 [13, 17].

#### 2.1.2. Potential Target Identification of CSCC and UC

The corresponding targets of the bioactive components in CSCC were imported into the DrugBank database (https://www.drugbank.ca/) [18]. Human genes associated with UC were identified from the GeneCards database (https://www.genecards.org/). Genes with target requirements of relevance score ≥ 1 were considered to be notable UC-related expression targets [19].

#### 2.1.3. Establishment of a Component-Target Network and Topological Analysis

The C-T network of CSCC against UC was constructed using Cytoscape v.3.7.0 software (http://www.cytoscape.org) [20]. Features, such as degree, closeness centrality, betweenness centrality, and lower average shortest-path length, were employed by the “network analyzer” tool to analyze the key targets with a higher degree than the median average for the establishment of a major C-T network of CSCC.

#### 2.1.4. UC-Related Target Network Establishment

The UC-related CSCC targets were identified and submitted to STRING for protein-protein interaction (PPI) network mapping (https://string-db.org) [17]. Next, predicted direct and functional target genes with high confidence scores (≥0.7) for PPIs from the imported UC gene STRING data were collected to establish a PPI network and visualized with Cytoscape [21].

#### 2.1.5. Gene Ontology and Pathway Enrichment Analysis

All the candidate components and genes were further assessed via Gene Ontology (GO) annotation and Kyoto Encyclopedia of Genes and Genomes (KEGG) pathway enrichment analysis (http://bioconductor.org/biocLite.R). The top 20 terms of highly enriched pathways were visualized and integrated for “component-target-pathway” network construction. The “Network Analyzer” of Cytoscape was utilized to identify the core nodes that have the maximum degrees in the network.

### 2.2. Experimental Validation

#### 2.2.1. Animals

BALB/c mice were purchased from Beijing Vital River Laboratory Animal Technology Co., Ltd. (Beijing, China). Eight-week-old male mice were used in all experiments. All mice were housed at 25°C with a 12 h dark/light cycle in the Laboratory Animal Center of the Academy of Military Medical Science (AMMS) of China. All mouse experiments were performed in accordance with the guidelines of the Laboratory Animal Center of AMMS and the Ethics Committee of Chinese PLA General Hospital. No mice were excluded, and randomization and blinding were not adopted for experimental allocation.

#### 2.2.2. Induction of UC Model and Rectal Administration

**Figure 1:**
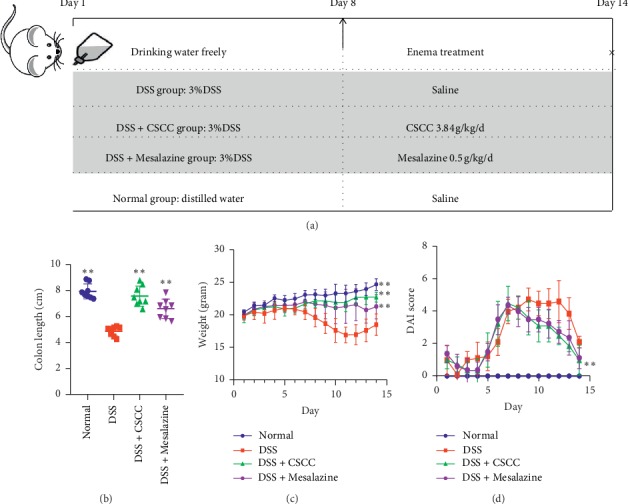
Protocol and effects of CSCC on DSS-induced colitis. (a) The protocol of colitis induction and CSCC treatment. (b) Colon length. (c) Body weight. (d) Disease activity index (DAI) score. Data are expressed as means ± standard deviation (*n* = 6). ^*∗*^*P* < 0.05, ^*∗∗*^*P* < 0.01 vs. DSS group.

The UC murine model was induced by the administration of 3% (w/v) dextran sulfate sodium (DSS; molecular weight: 36,000–50,000 Da; MP Biomedicals, USA) in distilled drinking water for 7 d, followed by distilled water for 7 d. Mice were allocated to normal, DSS, Mesalazine, and CSCC groups. Only the normal group did not receive DSS. The DSS, CSCC, and Mesalazine groups were treated with saline, CSCC (3.84 g/(kg·d), Coway, China, No. C1020171001), and Mesalazine (0.5 g/(kg·d), Luling Pharmaceutical, China, No. 071006), respectively, via rectal administration daily for 7 d (Figure 1(a)).

Body weight, stool consistency, and stool occult blood were monitored daily, and the data were calculated for disease activity index (DAI) with a grade scale of 0–3: loss of weight (0 = none, 1 = 1–5%, 2 = 6–10%, 3 > 10%), stool consistency (0 = normal, 1 = paste stools, 2 = loose stools, 3 = watery diarrhea), and stool occult blood (0 = normal, 1 = occult blood, 2 = bleeding, 3 = gross bleeding) [22, 23]. The colon length was recorded when all mice were sacrificed on the 15^th^ day for the collection of the colon and mesenteric lymph nodes (MLNs).

#### 2.2.3. Colon Real-Time Polymerase Chain Reaction (PCR) Analysis

Total RNA from the colon was prepared using TRIzol (Invitrogen) followed by reverse transcription to cDNA (Essence Biotech, China). Quantitative PCR (qPCR) was performed on a LightCycler® (Roche) with SYBR Green Supermix (ESscinece Biotech, China) in a 20 *μ*L reaction volume. The following primers were used: for IL-17A, 5′-TGATGCTGTTGCTGCTGCTGAG-3′ and 5′-CACATTCTGGAGGAAGTCCTTGGC-3′; for IL-10, 5′-CACTGCTATGCTGCCTGCTC-3′ and 5′-ACTGGGAAGTGGGTGCAGTT-3′; for IL-6, 5′-CTTCTTGGGACTGATGCTGGTGAC-3′ and 5′-AGGTCTGTTGGGAGTGGTATCCTC-3′; for TNF-*α*, 5′-GCCTCTTCTCATTCCTGCTTGTGG-3′ and 5′- GTGGTTTGTGAGTGTGAGGGTCTG-3′; for FoxP3, 5′-TTTCACCTATGCCACCCTTATC-3′ and 5′-CATGCGAGTAAACCAATGGTAG-3′; for IL-1*β*, 5′-GCTTCAGGCAGGCAGTATCACTC-3′ and 5′-TCTGCTGTCTGCTCTCAGTCCTC-3′; for TGF-*β*, 5′-CCAGATCCTGTCCAAACTAAGG-3′ and 5′-CTCTTTAGCATAGTAGTCCGCT-3′. The cycling program was set as follows: initial cycle of 95°C for 5 min, followed by 40 cycles of 95°C for 10 s and 60°C for 30 s. Relative mRNA levels were calculated by using the ∆∆Ct method using the equation 2^−∆∆Ct^.

#### 2.2.4. Enzyme-Linked Immunosorbent Assay (ELISA) for Cytokines

Colon segments were homogenized in phosphate-buffered saline (PBS) to remove excess blood, and the colon homogenates were centrifuged at 10,000 ×*g* for 5 min. The protein levels of IL-17A, IL-6, IL-1*β*, TNF-*α*, transforming growth factor-beta (TGF-*β*), and IL-10 were estimated using murine-specific ELISA assay kits (Abcam, USA) according to the manufacturer's instructions.

#### 2.2.5. Cell Isolation and Flow Cytometry

On the 15^th^ day, MLNs of mice were harvested by mechanical dissociation, and lamina propria mononuclear cells (LPMCs) were isolated from the colon, as described earlier [24]. Colons were cut and cleaned, and the epithelial layers were removed by incubation of the tissues in RPMI-1640 (Solarbio, China) containing 5 mM ethylenediaminetetraacetic acid (Solarbio, China) and 10 mM 4-(2-hydroxyethyl)-1-piperazine ethanesulfonic acid (Solarbio, China), supplemented with 10% fetal bovine serum (HyClone, USA) for 30 min at 37°C. LPMCs were isolated by first digesting pieces of colon segments in 1 mg/mL collagenase type IV, 0.05% DNase I, and 0.3% Dispase II (all from Sigma-Aldrich, USA) for 45 min at 37°C and then using 40–80% Percoll (GE, USA) gradient to purify the resulting cells. For flow cytometric analysis, cells from MLNs and LPMCs were stained with fluorochrome-conjugated monoclonal antibodies directed against the following cell surface antigens: Cluster of Differentiation 3e (CD3e) (100205), CD4 (553046), and CD25 (561048), and forkhead box P3 (FoxP3) (563101). All antibodies were purchased from BioLegend (USA). For the detection of cytokines in Th17 cells, the cells were activated for 6 h with Cell Activation Cocktail (with Brefeldin A) (BioLegend, USA). The cells were then fixed and permeabilized using a Fixation/Permeabilization Solution Kit (BD Bioscience, USA) and stained with antibodies directed against FoxP3 (563101) and IL-17A (506915). Flow cytometry data of MLNs and LPMCs were obtained using LSRII (BD Biosciences) and analyzed with FlowJo software.

#### 2.2.6. 16S rRNA Gene Sequencing and Microbiome Analysis

On the 9th, 12th, and 14th days, fresh stool samples were collected for 16S rRNA gene sequencing. Total bacterial genomic DNA was isolated using the DNA Stool Kit (Biomiga, Shanghai, China). A NanoDrop 2000 ultraviolet spectrophotometer (Thermo Fisher Scientific, USA) was used to detect the quality and quantity of extracted DNA. The 16S rDNA V3-V4 region was amplified by PCR and sequenced using an Illumina MiSeq PE300 system (MajorBio Co., Ltd., Shanghai, China) using universal primers 338F (5′-ACTCCTACGGGAGGCAGCAG-3′) and 806R (5′-GGACTACHVGGGTWTCTAAT-3′). The data were analyzed on the free online MajorBio Cloud Platform. Microbial association network construction within administration groups helped us explore the apparent correlations between cooccurring taxa. Data of cooccurrence analysis from MajorBio Cloud were entered into Cytoscape software for the generation of association networks of gut microbiome from multiple groups [25, 26].

## 3. Statistical Analysis

Data from statistical analysis were represented as means ± standard deviation. Multiple comparisons between groups were analyzed using one-way Analysis of Variance (ANOVA), and the comparison of two groups was performed using Student's *t*-test. *P* < 0.05 was considered to be statistically significant.

## 4. Results

### 4.1. Network Pharmacology-Based Analysis

#### 4.1.1. CSCC Component Identification

**Figure 2:**
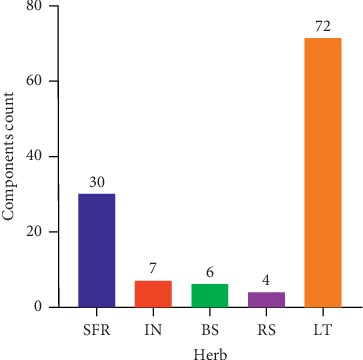
Candidate bioactive components in CSCC. The 119 candidate bioactive components (SFR = 30; IN = 7; BS = 6; RS = 4; LR = 72) of CSCC were determined with DL index ≥0.18, OB ≥ 30%, and Caco-2 ≥ 0.4.

The CSCC prescription consists of five herbal medicines, namely, SFR, IN, BS, RS, and LR. A total of 514 monomer components present in the five herbs of CSCC were obtained from the TCMSP database. Properties of the monomer components, including drug-likeness (DL) index ≥ 0.18, oral bioavailability (OB) ≥ 30%, and intestinal epithelial permeability (in Caco-2 cells) ≥ 0.4, were analyzed and screened to yield 119 bioactive components of CSCC (Table 1, Supplementary Table 1, and Figure 2).

#### 4.1.2. Target Identification of CSCC on UC

**Figure 3:**
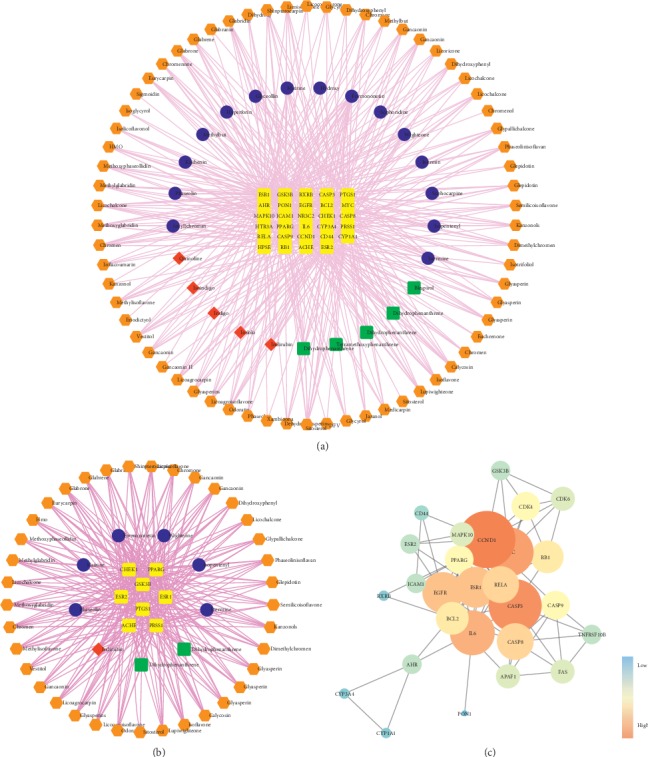
Network of CSCC against UC. (a) The component-target (C-T) network of CSCC against UC containing 120 nodes and 457 edges. (b) The major C-T network established with nodes with degrees higher than average node degrees of 5.022. The yellow rectangles represent targets; the blue, red, green, and orange rectangles represent the components from SFR, IN, BS, and LR, respectively. (c) The PPI network of target genes from CSCC against UC. The ellipse nodes represent targets, and the colors of nodes from blue to orange represent the descending order of degree values.

CSCC C-T relationship data (number = 665) were obtained for the 119 bioactive component candidates by using the DrugBank database, yielding 96 monomer components and 47 target genes; the details are shown in Supplementary Table 2. The GeneCards database was used to identify a total of 1949 human target genes associated with UC that met the requirements of relevance score ≥1 (Supplementary Table 3). Cytoscape software was used to establish the C-T network and identify the intersection of the 47 putative target genes of the candidate bioactive components in CSCC and 1949 UC-related target genes, which consisted of 29 targets of consensus genes as potential therapeutic targets of CSCC in the treatment of UC. The C-T network of CSCC containing 120 nodes (29 gene targets and 91 component targets) and 457 edges indicated all of the component-target interactions; detailed information is shown in Figure 3(a).

#### 4.1.3. Network Topological Analysis

The topological analysis was further carried out on the nodes in the C-T network of CSCC using the calculations of Cytoscape's NetworkAnalyzer tool on the main topological features (degree, betweenness centrality, average shortest-path length, and closeness centrality) [27, 28]. A major C-T network of CSCC was established with node degrees higher than an average node degree value of 5.022; the detailed information is shown in Figure 3(b). Eight major UC-related target genes with a higher degree (5.022), closeness centrality, betweenness centrality, and lower average shortest-path length (Supplementary Table 4) were identified along with 46 main bioactive components (Supplementary Table 5). Formononetin from SFR, Licochalcone A from LR, and Matrine from SFR had the highest degree, betweenness centrality, and average shortest-path length of 18, 0.192, and 3.692, respectively, which indicates that these three components occupied crucial positions in this network.

#### 4.1.4. Clustering Analysis

The 29 UC-related target genes regulated by CSCC were imported into the STRING database for the examination of PPIs. Calculating the interaction confidence scores of all related consensus target genes, the STRING database predicted 21 target genes and 6 predicted functional genes with high confidence (interaction score ≥ 0.7). Another PPI network with 27 nodes (21 target genes and 6 predicted functional genes) and 81 edges was generated after importing the data into Cytoscape (Figure 3(c)). From these genes, ten major targets of CSCC against UC were identified with a higher degree >6 (average target gene degree), namely, CCND1, CASP3, MYC, IL6, ESR1, EGFR, CASP8, RELA, RB1, and BCL2. The detailed results are shown in Supplementary Table 6.

#### 4.1.5. Pathway Enrichment Analysis

**Figure 4:**
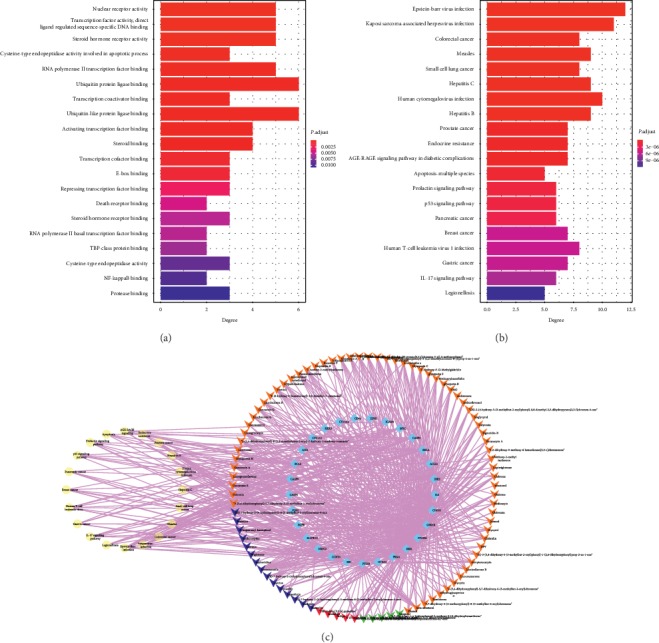
KEGG pathway for component-target pathway network analysis. Target genes were further subjected to Gene Ontology (GO) and Kyoto Encyclopedia of Genes and Genomes (KEGG) enrichment analysis. (a) The top 20 GO term categories with adjusted *P* values (*P* adjust) <0.05. (b) The top 20 enriched clusters of the KEGG pathway. (c) Component-target pathway network of CSCC. The light blue rhombuses represent targets; the yellow ellipses represent the pathways; the blue, red, green, and orange represent the components from SFR, IN, BS, and LR, respectively.

The 29 candidate target genes of CSCC against UC were further used to perform GO annotation and KEGG pathway enrichment. Sixty significantly enriched GO terms (adjusted *P* values (*P* adjust) <0.05) were detected on the basis of which CSCC modulates UC. The top five enriched GO terms are nuclear receptor activity, transcription factor activity, steroid hormone receptor activity, cysteine-type endopeptidase activity involved in the apoptotic process, and RNA polymerase II transcription factor binding. The top 20 terms are shown in Figure 4(a) and Supplementary Table 7. Ninety-seven KEGG pathways met the requirements of *P* adjust <0.05, and 20 enriched pathways, including endocrine resistance, apoptosis-multiple species, p53 signaling pathway, human T-cell leukemia virus 1 infection, and interleukin-17 (IL-17) signaling pathway, were identified as major pathways (Figure 4(b) and Supplementary Table 8). Network analysis was further conducted with the integrated results of the aforementioned top 20 KEGG pathways, bioactive components, and the predicted targets (Figure 4(c)). Through the calculations of the Cytoscape's NetworkAnalyzer on the features of degree, network analysis suggested that Kaposi sarcoma-associated herpesvirus infection, Formononetin from SFR, and ESR1 may be the core pathways, components, and target genes, respectively, involved in the activity of CSCC against UC (Supplementary Table 9).

### 4.2. Experimental Validation

#### 4.2.1. CSCC Inhibited the Development of DSS-Induced Colitis Murine Model

To validate the remission effect of CSCC against UC as predicted by network pharmacology analysis, a DSS-induced UC murine model was established [29]. Mice exhibited apparent changes, such as body weight and DAI score, on day 7 of DSS administration. The details of the development of the DSS-induced colitis murine model are shown in Figure 1(a). As expected, the administration of CSCC protected mice from colitis induced by DSS. The DAI scores gradually decreased from the 8^th^ day to the 10^th^ day in the CSCC and Mesalazine groups compared to the DSS group (*P* < 0.05). Furthermore, there were significant differences in the daily body weight loss in the CSCC, Mesalazine, and DSS groups after the administration of the enema treatment (*P* < 0.05). Compared to the normal group on the 14^th^ day, colon lengths of sacrificed mice showed a reduction of approximately 38.4%, 7.4%, and 17.6% in the DSS, CSCC, and Mesalazine groups, respectively (Figures 1(b)–1(d)).

#### 4.2.2. CSCC Inhibited Colitis Development via IL-17 Signaling Pathway

**Figure 5:**
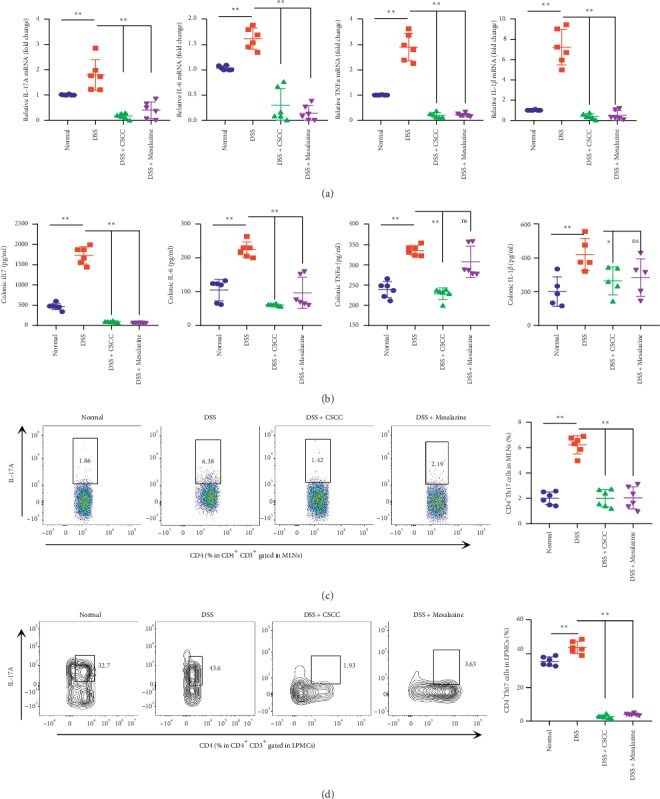
Effects of CSCC on levels of IL-17 pathway cytokines. CSCC attenuated DSS-induced colitis by suppressing of the IL-17 pathway. (a) The reduced protein levels of IL-17A and the cytokines it induces, namely, IL-6, IL-1*β*, and TNF-*α*. (b) Suppression of mRNA levels of the hallmark cytokines of the IL-17 pathway. (c-d) Representative color plots showing the flow cytometric analysis of Th17 cells isolated from MLNs and LPMCs, with quantification of the Th17 populations in the total CD4^+^ T-cell population. Data are expressed as means ± standard deviation (*n* = 6). ^*∗*^*P* < 0.05, ^*∗∗*^*P* < 0.01 vs. DSS group.

As an important UC-related inflammation pathway, the IL-17 signaling pathway was a major pathway predicted by the network pharmacology analysis of CSCC against UC [30–32]. Although IL-17 signaling confers protection against extracellular pathogens in the host, the IL-17 family of cytokines plays a crucial role in the inflammatory pathology of autoimmune diseases, with a close correlation with the subset of T helper 17 (Th17) cells and their hallmark cytokine, IL-17A [33–35]. Further experimental validation revealed that CSCC attenuated DSS-induced colitis by the suppression of the mRNA levels of IL-17A and of the cytokines it induces, such as IL-6, IL-1*β*, and TNF-*α*. In agreement with mRNA data, ELISA results showed that the protein levels of the aforementioned cytokines decreased significantly in the CSCC group (*P* < 0.05 vs. DSS). Th17 cells, the main cells secreting IL-17A, were obviously downregulated in CSCC and Mesalazine groups (*P* < 0.01 vs. DSS). These results suggest that CSCC suppressed Th17 cell-driven autoimmune inflammation via regulation of the signaling by IL-17A, IL-6, IL-1*β*, and TNF*α* (Figures 5(a)–5(d)).

#### 4.2.3. CSCC Impacts the IL-17 Pathway by Regulating Gut Microbiota

**Figure 6:**
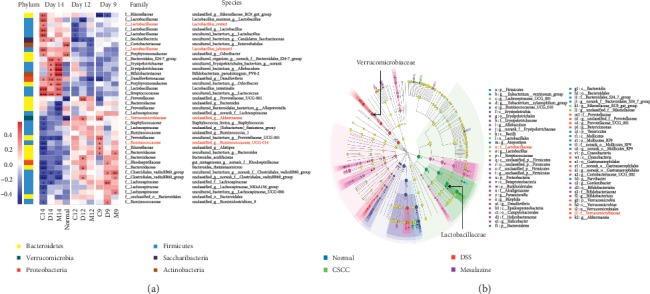
The role of CSCC in regulating the microbiome. (a) The heatmaps of correlation analysis between the dominant genus and rectal administration on the 9^th^, 12^th^, and 14^th^ days. The degree of correlation is expressed in color grade. (*n* = 6, ^*∗*^*P* < 0.05, ^*∗∗*^*P* < 0.01). (b) Phylogenetic trees for administration in association with overlapping microbiota features. Green represents higher relative abundance in CSCC, and red represents DSS groups.

**Figure 7:**
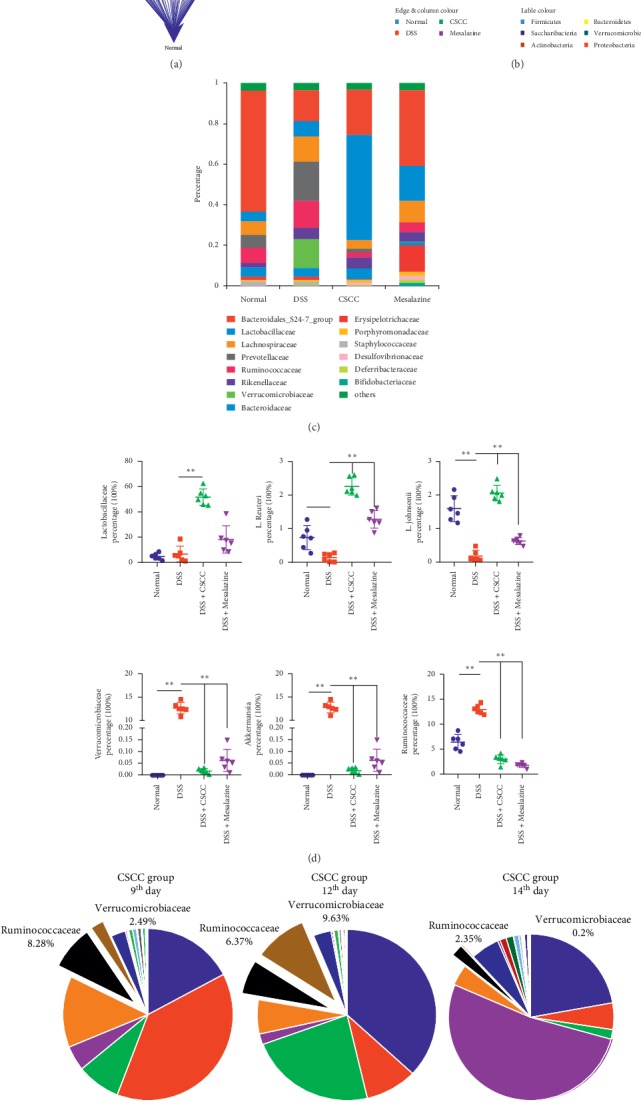
Effect of treatment on the microbiome of DSS-induced colitis. (a-b) Cooccurrence network of gut microbiome, on the 14^th^ day, in association with multiple groups and CSCC group. (c) Abundance and percentage of the different bacterial families in each group. The most abundant family was *Lactobacillaceae* in the mice that received CSCC enema treatment. (d) Differences in abundance of *Lactobacillaceae*, *Verrucomicrobiaceae,* and *Ruminococcaceae* (family levels), *L. reuteri*, *L. johnsonii*, and *Akkermansia* (species levels) in different groups. Data are expressed as means ± standard deviation (*n* = 6). ^*∗*^*P* < 0.05, ^*∗∗*^*P* < 0.01 vs. DSS group. (e) The relative populations of *Verrucomicrobiaceae* and *Ruminococcaceae* in the CSCC group at multiple time points.

Several lines of evidence support a critical role for infectious gastrointestinal microbes in the dysregulated immune system, leading to the differentiation of naive T cells into effector Th17 cells and the production of IL-17, TNF-*α*, and IL-6 [25, 36]. To determine the causality between gut microbiota and the CSCC-mediated inhibition of the development of colitis, continuous fecal samples collected from mice on the 9^th^, 12^th^, and 14^th^ days were submitted for bacterial microbiota profiling. The heatmaps shown in Figure 6(a) illustrate the relationships among the different groups; four positive microbial species showed a significant correlation with CSCC on the 14^th^ day, with a tendency for microbiota regulation due to the daily rectal administration. *Akkermansia* and Ruminococcaceae UCG-014 showed a negative correlation with CSCC, whereas *Lactobacillus reuteri* and *Lactobacillus johnsonii* showed a positive correlation. Specific microbiota features that are associated with the administration and that could be replicated at the genus level in any of the groups were further analyzed (Figure 6(b)). Comparison of relative abundance in DSS and CSCC groups revealed differences in concordance at the family level (Verrucomicrobiaceae and Lactobacillaceae). The histograms in Figure 7(c) show the top 15 phyla in four groups and reveal the gut microbiota community structures and relative abundance. As shown in Figure 7(d), the relative abundance of Lactobacillaceae, Verrucomicrobiaceae, and Ruminococcaceae (family levels) and of *L. reuteri*, *L. johnsonii*, and *Akkermansia* (species levels) was significantly different in the CSCC group compared to that of the DSS group. The most abundant family was Lactobacillaceae in mice that received CSCC enema treatment. The bacterial features based on sample relative abundance also revealed a depression tendency of Ruminococcaceae. And the relative population of Verrucomicrobiaceae was the lowest on the 14^th^ day but peaked on the 12^th^ day (Figure 7(e)). We then used all gut microbiota (at the family level) from different groups to execute cooccurrence network analysis among the different microbial communities (Figures 7(a)-7(b)). We found that CSCC administration possessed the highest “topological coefficient” score, suggesting a tendency for CSCC to have more shared bacterial species functions in the network, especially for Lactobacillaceae (Figure 7(b) and Supplementary Table 10).

#### 4.2.4. Inhibition of the IL-17 Signaling Pathway by CSCC Was Mediated by Treg Cells

**Figure 8:**
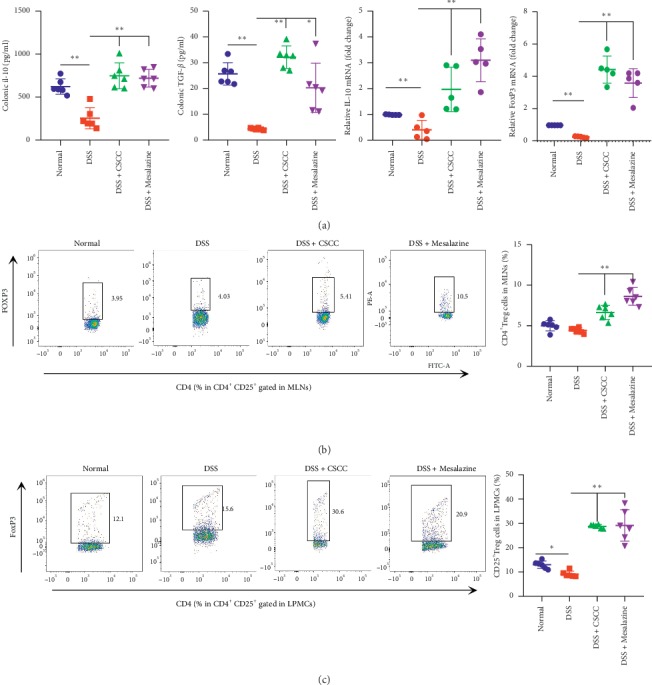
Effects of CSCC on levels of Treg cells. (a) The elevated protein levels of cytokines expressed by Treg cells, namely, IL-10 and TGF-*β*, and mRNA levels of IL-10 and FoxP3. (b–c) Representative color plots showing the flow cytometric analysis of Treg cells isolated from MLNs and LPMCs, with quantification of the Treg populations in the total CD4^+^ T cells. Data are expressed as means ± standard deviation (*n* = 6). ^*∗*^*P* < 0.05, ^*∗∗*^*P* < 0.01 vs. DSS group.

Evidence-based research has revealed a crucial role for the immune balance maintained by CD4^+^ T-cell differentiation into Th17 and Treg cells through the secretion of cytokines; for example, secretion of IL-10 and TGF-*β* promotes the differentiation of CD4^+^ T cells into Treg cells and attenuates the progress of colitis promoted by the IL-17 signaling pathway [37, 38]. Flow cytometry analyses were performed to detect the quantities of Treg cells in MLNs and LPMCs. Figures 8(c)-8(d) show that the numbers of Treg cells were markedly higher after the administration of CSCC and Mesalazine, as compared to the DSS group. In agreement with these flow cytometry results, the levels of the hallmark cytokines of Treg cells, IL-10 and TGF-*β*, were also significantly higher after rectal administration, at both the mRNA and protein levels (*P* < 0.01 vs. DSS group, Figures 8(a)-8(b)). These data show that the inhibition of the IL-17 signaling pathway by CSCC may be mediated by the induction of Treg cells.

## 5. Discussion

UC is a chronic gastrointestinal inflammation involving the rectal and colonic mucosa, with multifactorial pathogenesis that acts synergistically, such as immune system dysregulation and expansion of infectious gut microbiota [2, 39, 40]. The rectal administration of CSCC in the treatment of active UC has been successfully applied in TCM clinical practice for decades. By adopting the OLCM technique, CSCC has been shown to provide therapeutic efficacy in the treatment of active UC that is comparable to that of Mesalazine enteric-coated tablets in several randomized clinical trials [10, 11]. According to Chinese herbal medicine, CSCC consisting of five herbal medicines performs the functions of clearing damp-heat, cooling blood for hemostasis, detumescence for promoting granulation, and relieving spasm and pain. Based on the efficacy of CSCC in alleviating DSS-induced colitis in a mouse model, pharmacological activities of five herbal medicines in CSCC were further explored in terms of the multiple components and targets identified using a network analysis approach. We identified 29 consensus genes and the top 20 signaling pathways that could be modulated by CSCC, as predicted by network pharmacology data, which may be the potential therapeutic targets for UC. The IL-17 signaling pathway, a major enriched pathway of CSCC against UC, performs a crucial role in UC-related inflammation; hence, it was selected as a candidate for further investigation of its effector T cells and important hallmark cytokines, as well as the upstream factors, such as intestinal flora [41, 42].

Components of CSCC with DL ≥ 0.18, OB ≥ 30%, and Caco-2 ≥ 0.4 were considered as candidate bioactive components that could reach intracellular targets through the intestinal epithelial cell barrier. In the current study, a C-T network of 91 bioactive components and 29 targets was constructed for CSCC against UC, of which Formononetin from SFR as well as licochalcone A from LR may play important roles in the therapeutic effects of CSCC on UC with high degrees of 18, 12, and 10, respectively. Formononetin, the most significant component in CSCC, is a natural isoflavone found in many Chinese medicinal herbs, such as SFR, and could offer protection against acute colitis in DSS-induced mouse models through the inhibition of cellular signaling cytokines produced by T cells in intestinal inflammation [43, 44]. Licochalcone A obtained from LR attenuate DSS-induced colitis through the inhibition of NF-*κ*B, a key factor in the increase in the number of Th17 cells, and of IL-17 signaling [45, 46]. Several results provide vital evidence to support the conclusion of C-T network analysis and suggest a potential mechanism underlying the effects of CSCC treatment on UC.

To understand UC better, KEGG pathway analysis was combined with the targets and components of CSCC to perform network analysis and identified IL-17 signaling as a major enriched pathway; IL-17 signaling is characterized as one of the vital mechanisms in UC progression [24]. We found that IL-17 was mainly activated and produced from the Th17 subset of CD4^+^ T cells, which induce intestinal inflammatory responses and tissue damage by promoting the secretion of cytokines such as IL-6, TNF-*α*, and IL-1*β*. Importantly, the influence of gut microbiota was sufficient to enhance IL-17 expression in the gut by the release of IL-6 and IL-1*β*. These hallmark factors also exert an important effect in promoting IL-17 transcription, which is released from myeloid cells during the whole response to gut microbes [47–50]. However, further investigation is required to determine the colonic immune regulatory mechanisms of CSCC that modulate gut flora, thereby preventing IL-17 signaling. In the present study, we observed that BALB/c mice developed acute colitis and weight reduction occurred at day 8–12 after DSS treatment with a low concentration in order to maintain the gut inflammatory situation without extensive weight loss or even death [51]. And CSCC suppressed the IL-17 signaling pathway and its effector Th17 cells, in both MLNs and LPMCs, as well as its key cytokines, IL-17A, IL-1*β*, TNF-*α*, and IL-6 at mRNA and protein levels in the colonic tissue. These results suggest that the IL-17 signaling pathway is of crucial relevance to the therapeutic efficacy of CSCC.

Next, we selected three time points (9^th^, 12^th^, and 14^th^ days) to collect fecal samples to determine how CSCC influenced the gut microbiota of a DSS-induced colitis mouse model. The CSCC group showed a link between regulation of the gut microbiota after the daily rectal administration and the attenuation of immune dysregulation and DSS colitis. Our results show the continuous differences in colonic microbiota in DSS mice after the administration of CSCC enema, as evidenced by increased *L. reuteri and L. johnsonii* (Lactobacillaceae family) and decreased *Akkermansia* and Ruminococcaceae UCG-014. Notably, Lactobacillaceae were the most abundant family in DSS mice that received CSCC enema treatment, and they had more shared bacterial neighbors that probably functioned synergistically, according to the network analysis of the gut microbiome. This is consistent with the notion that Lactobacillaceae are a well-known anti-inflammatory microbial family that can restore the protective gut microbiome in DSS mice and cause downregulation of Th17 cells and differentiation of Treg cells in intestinal epithelial cells, thereby preventing cytokine-induced inflammation and ameliorating the severity of colitis symptoms in experimental models [52–54]. *Akkermansia*, a key species of Verrucomicrobiaceae, was found to exacerbate colonic damage due to its enzymatic capacity to degrade the mucus layer [40, 55]. The phylogenetic analysis of microbiota associated with the administration of the different treatments revealed that *Akkermansia* was a distinct taxon in the DSS group. Additionally, counts of *Akkermansia* were reported to increase markedly after DSS administration and contributed to an increase in gut permeability, thus triggering IL-17 signaling and the release of IL-6 and IL-1*β* by myeloid cells [56]. In addition, Ruminococcaceae UCG-014 may be a key taxon in Crohn's Disease and UC and is associated with higher levels of proinflammatory cytokines in colitis models [25, 57, 58].

The present study suggests that the administration of CSCC led to gut microbiota recovery and intestinal immune homeostasis in DSS-induced colitis. Data on immune regulation have provided intriguing clues on the restoration of Th17/Treg balance, which was reciprocally regulated by Treg cell mediators, such as TGF-*β* and IL-10. There are compelling data that indicate that the formation of Treg cells may be promoted by certain anti-infectious gut flora, such as Lactobacillaceae, Bifidobacteriaceae, and Rikenellaceae, which can regulate proinflammatory pathways by reducing cytokines, such as IL-17A and TNF-*α*, inducing immune tolerance, and suppressing effectors of T cells [59–61]. Consistent with these findings, the CSCC group in the present study has shown an increased percentage of Tregs, which could be the reason for the reduced expression of IL-17 signaling cytokines.

Taken together, the pharmacological mechanism by which CSCC inhibited UC was confirmed through multiple approaches such as network pharmacology, gastrointestinal microbiome analysis, and experimental validation. We identified the bioactive components and potential targets and pathways of CSCC against UC progression. Further investigation of gut microbiota and hallmark cytokines in the IL-17 signaling pathway may help identify the mechanism of the inhibitory effects of CSCC on UC. Our study demonstrates the therapeutic efficacy of CSCC, a herbal formula, in UC treatment and shows the effectiveness of a network pharmacology approach in exploring the mechanisms of herbal medicine.

## Figures and Tables

**Table 1:** Number of components in CSCC with OB ≥ 30%, DL index ≥ 0.18, and Caco-2 index ≥ 0.4.

Herbs	Total	OB ≥ 30%	OB ≥ 30% and DL ≥ 0.18	OB ≥ 30%, DL ≥ 0.18, and Caco-2 ≥ 0.4
SFR	113	56	45	30
IN	29	17	9	7
BS	36	12	9	6
RS	41	15	13	4
LT	295	143	92	72

## Data Availability

The data of this study are included within the article and Supplementary Tables.

## Supplementary Materials

Supplementary Materials Supplementary Table 1: the selected bioactive components (DL index≥ 0.18, OB ≥ 30%, and Caco-2 ≥ 0.4) in CSCC for analysis obtained from TCMSP database. Supplementary Table 2: the summary of protein targets from the candidate bioactive components of CSCC in the DrugBank database. Supplementary Table 3: the detailed information of UC-related human targets (relevance score≥ 1) collected from GeneCards database. Supplementary Table 4: the topological parameters of major UC-related target genes with higher degree than average (degree = 5.022). Supplementary Table 5: the topological parameters of major bioactive components in CSCC with node degrees higher than average (degree = 5.022). Supplementary Table 6: the topological parameters of PPI network obtained from STRING database. Supplementary Table 7: the detailed information of enriched CSCC-related GO annotations (*P* adjust< 0.05). Supplementary Table 8: the detailed information of enriched CSCC-related KEGG pathways (*P* adjust< 0.05). Supplementary Table 9: the topological parameters of component-target-pathway network through Cytoscape Network analyzer's calculation. Supplementary Table 10: the topological parameters of gut microbiota in the family level through Cytoscape Network analyzer's calculation.
